# Anti-Prion Screening for Acridine, Dextran, and Tannic Acid using Real Time–Quaking Induced Conversion: A Comparison with PrP^Sc^-Infected Cell Screening

**DOI:** 10.1371/journal.pone.0170266

**Published:** 2017-01-17

**Authors:** Jae Wook Hyeon, Su Yeon Kim, Sol Moe Lee, Jeongmin Lee, Seong Soo A. An, Myung Koo Lee, Yeong Seon Lee

**Affiliations:** 1 Division of Zoonoses, Center for Immunology & Pathology, National Institute of Health, Korea Centers for Disease Control and Prevention, Chungcheongbuk-do, Korea; 2 Gachon BioNano Research Institute, Gachon University, Gyeonggi-do, Korea; 3 College of Pharmacy, Chungbuk National University, Cheongju, Korea; Deutsches Zentrum fur Neurodegenerative Erkrankungen, GERMANY

## Abstract

Prion propagation is mediated by the structural alteration of normal prion protein (PrP^C^) to generate pathogenic prion protein (PrP^Sc^). To date, compounds for the inhibition of prion propagation have mainly been screened using PrP^Sc^-infected cells. Real time–quaking-induced conversion (RT-QuIC) is one alternative screening method. In this study, we assessed the propagation inhibition effects of known anti-prion compounds using RT-QuIC and compared the results with those from a PrP^Sc^-infected cell assay. Compounds were applied to RT-QuIC reactions at 0 h or 22 h after prion propagation to determine whether they inhibited propagation or reduced amplified aggregates. RT-QuIC reactions in presence of acridine, dextran sulfate sodium (DSS), and tannic acid inhibited seeded aggregation with sporadic Creutzfeldt-Jakob disease at 0 h. After treatment at 22 h, amplified fluorescence was decreased in wells treated with either acridine or tannic acid. Compound activities were verified by western blot of RT-QuIC products and in a dye-independent conversion assay, the Multimer Detection System. Protease K-resistant PrP^Sc^ fragments (PrP^res^) were reduced by DSS and tannic acid in the PrP^Sc^-infected cell assay. Importantly, these inhibitory effects were similar despite different treatment times (0 h versus 3 days). Consequentially, RT-QuIC enabled the more specific classification of compounds according to action (i.e., inhibition of prion propagation versus reduction of amplified aggregates). RT-QuIC addresses the limitations of cell-based screening methods and can be used to further aid our understanding of the mechanisms of action of anti-prion compounds.

## Introduction

Prion diseases are fatal neurodegenerative diseases that trigger the accumulation of pathogenic prion protein (PrP^Sc^) and neuronal death in humans and animals [1]. The process of prion propagation involves the structural alteration of host-encoded cellular prion protein (PrP^C^) to PrP^Sc^ and the autocatalytic amplification of pathogenic protein [2]. PrP^Sc^ is largely protease-resistant, insoluble, β-sheet rich, and capable of aggregation as a hallmark of prion disease [3]. Therefore, inhibiting the conversion of PrP^C^ to PrP^Sc^ and/or facilitating the degradation of PrP^Sc^ are primary strategies for anti-prion pharmaceutical development.

Previous studies have investigated pharmacotherapy, immunotherapy, and cell therapy approaches to prion disease [4, 5]. In recent years, most novel anti-prion compounds have been screened and validated using permanent PrP^Sc^-infected cell models [6–8]. In this assay, cells are plated (1 × 10^5^ cells/well), allowed to stably attach, and achieve 50% confluence within 24 h. Attached cells are then incubated with anti-prion compounds for 5–8 days. Then, within 2 days, protease K-resistant PrP^Sc^ fragments (PrP^res^) are detected by western blotting [9–11]. Although cell screening is mandatory to evaluate the actions of drugs in the cellular environment *in vitro*, this method does not differentiate between the abilities of potential anti-prion compounds to inhibit prion propagation and degrade PrP^res^ aggregates.

Real time–quaking-induced conversion (RT-QuIC) is a recent innovation that allows the real-time detection and monitoring of the conversion of a recombinant PrP (rPrP) to synthetic PrP amyloid fibrils [12]. RT-QuIC has been successfully used to detect small amounts of PrP^Sc^ in brain tissue, cerebrospinal fluid, blood, saliva, and nasal fluid from human, cervid, ovine, bovine, and rodent species [13–18]. Amplified PrP^res^ resultant from the conversion of rPrP to its aggregated form is shown by increased Thioflavin T (ThT) fluorescence, which directly indicates the formation of beta-sheet structures in PrP^res^ aggregates. Here, we hypothesized that synthetic prion propagation in a RT-QuIC reaction seeded with PrP^Sc^-infected brain homogenate would be inhibited by anti-prion compounds and easily monitored using fluorescence. Moreover, this reaction system would enable differentiation between anti-prion compound inhibition of propagation and aggregate degradation.

Acridine, dextran sulfate sodium (DSS), and tannic acid each have alleged anti-prion activity and were selected from previous reports where they were used for the treatment of other diseases or symptoms including malaria, thrombosis, and oxidation, respectively. The goal of this study was to evaluate the usefulness of RT-QuIC for screening these anti-prion compounds by comparing the results of compound activities indicated by RT-QuIC and PrP^Sc^-infected cell screening methods.

## Materials and Methods

### Compounds

All compounds (acridine orange hydrochloride [318337], DSS [67578], tannic acid [403040], quinacrine dihydrochloride (Q3251) and doxycycline hyclate (D9891) were purchased from the same vendor (Sigma-Aldrich, Saint Louis, MO, USA) in order to ensure consistency in quality. Quinacrine and doxycycline were used as controls. Compounds were dissolved in distilled water or dimethyl sulfoxide (DMSO) to produce 50 mM stock solutions and stored at −80°C until use. The class, medical use, and solubility of each test compound was surveyed from manufacturer reports and the ChemSpider database (Table 1) [19–21].

**Table 1:** Compounds information.

Name	Product name	Class	Medical use	Soluble	Reference
Acridine	Acridine Orange hydrochloride	Tricyclic compound	Antimalarial	Water	[19]
DSS	Dextran sulfate sodium salt	Polyanionic compound	Thrombosis	Water	[22]
Tannic acid	Tannic acid	Polyphenolic compound	Antioxidation	Water	[21]

### Human biological samples

Brain homogenate from a confirmed case of sporadic Creutzfeldt-Jakob disease (sCJD) was obtained from the National Institute for Biological Standards and Control (NIBSC; reference code NHBX0/0001). Homogenate was serially diluted 10-fold from 10^-2^ to 10^-7^. CSF samples were obtained from Korean patients with confirmed sCJD (3 patients) and from patients with progressive dementia (24 patients) as part of routine diagnoses. A CSF sample from a patient with Alzheimer’s disease (AD) (1 patient) was provided by Dr. Kim at Seoul National University Bundang Hospital in Korea. This study was approved by the Institutional Review Board (IRB) of the Korea Centers for Disease Control and Prevention (IRB No. 2014-06EXP-04-R-A). Written informed consent was obtained from the patients or their legal guardians.

### RT-QuIC reaction

Human rPrP (residues 23–231; accession no. K02234) was produced and the RT-QuIC reaction was performed as per Wilham et al. and Cramm et al. with minor modifications [12, 23]. The final composition of the reaction buffer was as follows: 162 mM phosphate buffer (pH 6.9), 170 mM NaCl, 1 mM EDTA, 10 μM ThT, and 0.1 mg rPrP. Ninety-six microliters of buffer was dispensed into each well of a clear-bottomed black 96-well microplate (Nunc, 265301). Two microliters of diluted sCJD brain homogenate was seeded into each well. Compounds were applied at different times in order to examine compound effects in the presence or absence of aggregation. Treatment at t = 0 h was a condition in which there was not considered to be any aggregation and treatment at t = 22 h was a condition in which there was considered to be a sufficient amount of aggregation. These experiments were performed in order to determine whether a given compound inhibited the conversion of PrP^C^ to PrP^res^ or eliminated amplified PrP^res^ aggregates.

For treatment at 0 h, 2 μL of each compound (acridine, DSS, tannic acid, or doxycycline at 20 μM and quinacrine at 5 μM) was added to wells at t = 0 h. For treatment at 22 h, fluorescence saturation at 65,000 relative fluorescence units (rfu) was confirmed after 22 h and subsequently 2 μL of each compound was added to wells (all compounds were applied at the same concentration). DMSO treatment was used as a negative control. The plate was sealed and incubated for 90 h at 44°C with intermittent shaking using the BMG Labtech Optima Fluostar plate reader. Each reaction was performed in quadruplicate. A positive response was denoted if the average of the 2 highest readings at 72 h was greater than 2-fold of the negative control, as described in previous studies using saturation at 65,000 rfu [23, 24]. To ensure optimal RT-QuIC conditions in our laboratory, we repeatedly validated the cut-off in CSF samples from 24 progressive dementia patients, serially diluted sCJD homogenate, and sCJD or AD CSF-seeded reactions.

### ScN2a cells

ScN2a cells were derived from the neuroblastoma-2a cell line obtained from the American Type Culture Collection and persistently infected with PrP^Sc^ (Rocky Mountain Laboratory [RML] strain). This cell line was generously provided by Dr. Ryu at Hanyang University in Korea. Cell culture was performed as described previously [25]. Briefly, cells were seeded in a T25 cm^2^ flask (TPP, Z707481) at 2 × 10^5^ cells/flask and pre-incubated in Dulbecco’s Modified Eagle Medium (Gibco, 12430) containing 10% fetal bovine serum, 1% penicillin-streptomycin (Gibco, 15140), and 2 mM l-glutamine (Gibco, 25030) for 24 h under 5% CO_2_ at 37°C.

We tried to mimic different PrP^res^ conditions in cells, similar to the RT-QuIC reactions. When compound was applied at 0 h, the cells had achieved about 50% confluence and were considered able to endure a non-toxic concentration of the compound. PrP^res^ was not maximally produced from PrP^C^ at this point in time; that is, minimal PrP^C^ had been converted into PrP^res^. Treatment at 0 h was therefore able to test whether a given compound interfered with the conversion of PrP^C^ to PrP^res^ in cells. When compound was applied at 3 days, the cells had achieved about 98% confluence. Therefore, PrP^res^ production was not acute and we assumed that all PrP^C^ had been converted into mature PrP^res^. These experiments were used to confirm whether a compound inhibited the conversion of PrP^C^ to PrP^res^ or eliminated amplified PrP^res^ aggregates.

For treatment at 0 h, pre-incubation media was replaced with fresh media containing test compound. After incubation for 3 additional days, the media was replaced again with fresh media containing test compound. For treatments at 3 days, pre-incubation was initially replaced with fresh media. After incubation for 3 additional days, the media was replaced with fresh media containing test compound. After total incubation for 6 days, media was removed, the cells were washed with PBS, and the remaining cells were lysed. Control experiments were treated with DMSO vehicle (0.1% v/v). Each compound was tested in duplicate in 3 independent experiments.

### Cytotoxicity assay

A MTT-based cytotoxicity assay was performed as described previously [25]. Compounds applied at 5 different concentrations between 1 and 100 μM or DMSO vehicle were incubated with cells for 3 days. Cytotoxicity was expressed as the percentage of the signal observed in DMSO-treated control cells.

### Western blot analysis

For the detection of PrP^res^ in RT-QuIC products, 50 μL of solution was collected from wells treated with acridine, DSS, tannic acid, doxycycline, or DMSO vehicle upon termination of the RT-QuIC reaction. The solutions were digested with proteinase K (PK) (20 μg/mL) (Merck, 70663) for 60 min at 37°C, stopped with 2 mM Pefabloc (Roche, 11429876001), and centrifuged for 90 min at 20,000 × *g* at 4°C. Pellets were resuspended in SDS sample buffer (125 mM Tris-HCl, pH 6.8, 5% (vol/vol) glycerol, 6 mM EDTA, 5% (wt/vol) SDS, 0.04% (vol/vol) bromophenol blue, and 12.5% (vol/vol) β-mercaptoethanol) and separated by SDS-PAGE. Proteins were probed with the monoclonal PrP antibody, 6H4 (1:2,500) (Prionics, PRN-01-011).

Cells were rinsed once in phosphate-buffered saline (PBS) and lysed in ice-cold lysis buffer (10 mM EDTA, 10 mM Tris, pH 8.0, 100 mM NaCl, 0.5% [wt/vol] Nonidet P-40, and 0.5% [wt/vol] sodium deoxycholate). Lysates were sonicated at an amplitude of 30%. The total protein concentration was adjusted to 1 mg/mL. Subsequent PK digestion and western blotting were performed as mentioned above. Signals were visualized using ECL (Elpis Biotech, EBP-1073) and protein bands were scanned using an Image Scanner III (GE). Another blot of the cell lysates without PK digestion was probed using a β-actin antibody (1:5000) (Cell Signaling, 4970). The relative band densities are shown as the volume intensity/mm^2^ relative to the β-actin band density.

### MDS assay

The MDS assay is a dye-independent conversion assay that has been used previously to screen anti-prion compounds [25]. The MDS assay kit was supplied by People Bio Inc. and performed according to manufacturer specifications with minor modifications [26]. Briefly, test compounds (0.05, 0.2, 1, 5, and 20 μM) were mixed with the reaction buffer containing 50 ng of recombinant PrP, 1% Triton X-100, 10% Blockace, and Tris-buffered saline containing 0.1% (vol/vol) Tween 20 (TBST) in 2 mL screw cap tubes. Doxycycline (0.05, 0.2, 1, 5, and 20 μM), quinacrine (1 and 5 μM), and DMSO (0.1% vol/vol) were used as negative, positive, and vehicle controls, respectively. The mixture was incubated with continuous shaking for 3 h at 37°C. 3E7 PrP antibody (2 μg) conjugated to magnetic beads and HRP-conjugated PrP T2 antibody (8 μg) were added to the pre-incubated mixture. After additional incubation for 1 h under the same conditions, the beads were separated and washed 3 times with TBST using a magnetic particle concentrator (Invitrogen, 120.20D). Luminescence signal was developed by adding Supersignal ELISA pico chemiluminescence substrate (Pierce, 37070) and quantified using a VICTOR3 microplate reader (Perkin Elmer, 1420–032).

### Statistical analysis

Each experiment was repeated 3 times. A 1-way analysis of variance with Tukey-Kramer *post hoc* tests was conducted using Microsoft Excel. Differences were considered to be statistically significant when *P* < 0.05.

## Results

### Determination of optimal RT-QuIC condition

**Fig 1:**
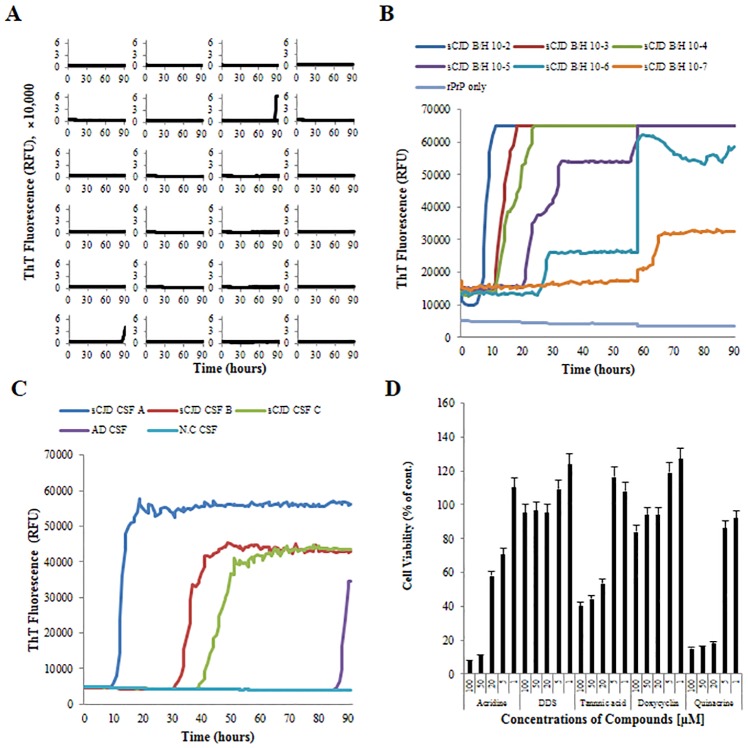
Optimization of RT-QuIC and determination of compound cytotoxicity. Time point optimization was performed using CSF samples from 24 cases of progressive dementia (A) and validation was performed using serially diluted sCJD brain homogenate (B) and CSF from a sCJD or AD patient (C). Cell viability was assessed in the presence of 5 different compounds at 5 different concentrations between 1 μM and 100 μM (D). Values represent the mean of 3 independent experiments and the standard deviation is shown as error bars, *P* < 0.05.

We optimized the RT-QuIC conditions prior to test compound screening. For a negative control, CSF samples from progressive dementia patients without sCJD (n = 24) were tested. Nonspecific self-aggregation was identified in 2 of 24 samples at more than 85 h (Fig 1A). For a positive control for brain seed, sCJD homogenates were serially diluted 10-fold from 10^-2^ to 10^-7^ and tested. ThT fluorescence was acutely increased in a concentration-dependent manner (Fig 1B). For a positive control for CSF, samples from sCJD and AD patients were tested. Fluorescence was increased in all sCJD CSF samples, and fluorescence in the AD CSF sample was increased after 85 h (Fig 1C). Accordingly, a 72 h time point was deemed to be reasonable and adopted for further study.

### Determination of compound concentrations for treatment

A MTT cytotoxicity assay was used to determine appropriate treatment concentrations for RT-QuIC and cell screening. Acridine was highly toxic at > 50 μM, tannic acid was non-toxic at < 20 μM, DSS and doxycycline permitted cell viability in excess of 90% at 100 μM. Quinacrine showed consistent toxicity (high toxicity at > 5 μM) as per a previous study [25]. Final concentrations between 1 and 20 μM were found to be optimal for treatment, and therefore, concentrations of 5 and 20 μM were selected for further study (Fig 1D).

### RT-QuIC screening

**Fig 2:**
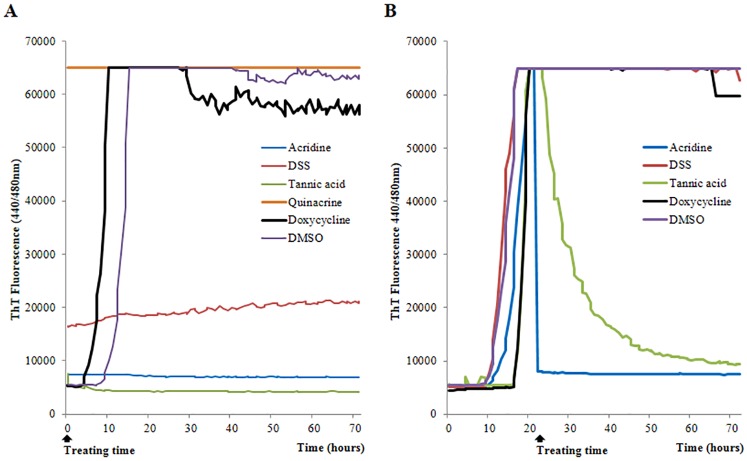
Inhibitory effects in RT-QuIC screening. Compounds were applied to RT-QuIC reactions at 0 h (A) and 22 h (B). Each sample was performed in quadruplicate. The average ThT fluorescence value of the 2 highest readings at 72 h per well was plotted against time. DMSO vehicle was used as a negative control.

Acridine, DSS, tannic acid, and doxycycline were applied to RT-QuIC reactions at the specified times. Test wells that were treated with quinacrine were saturated at 65,000 rfu at the first reading and thus, were unable to show further amplification. Moreover, a visible yellow fluorescent color similar to that of ThT was observed during the preparation of stock solutions. Therefore, quinacrine was excluded from the following studies. Inhibition of prion propagation was observed at the initial reaction after treatment at 0 h. Increases in fluorescence were inhibited in wells that were treated with acridine or tannic acid compared to those treated with doxycycline or DMSO over the 72-h period. DSS treatment produced a slight fluorescence elevation that was less than 2-fold between the first and last readings (which was our criterion for a positive response) over the 72-h period. Alternatively, fluorescence was elevated to saturation in wells without any compound between 5 and 20 h. Thus, we confirmed that the generation of synthetic PrP aggregates was inhibited by acridine, DSS, and tannic acid (Fig 2A). Additionally, the elimination of fully aggregated PrP amyloid was observed after treatment at 22 h. Fluorescence reached saturation in untreated wells between 12 and 20 h. Thereafter, acridine, DSS, and tannic acid were added to the wells. Acridine and tannic acid reduced fluorescence to baseline levels during the total reaction. In particular, acridine took less time than tannic acid to reduce fluorescence to baseline level. DSS did not produce an observable fluorescence change and was comparable to the negative control. Thus, we confirmed that PrP aggregates were reduced by acridine and tannic acid treatments (Fig 2B).

### Confirmation of PrP^res^ in RT-QuIC products

**Fig 3:**
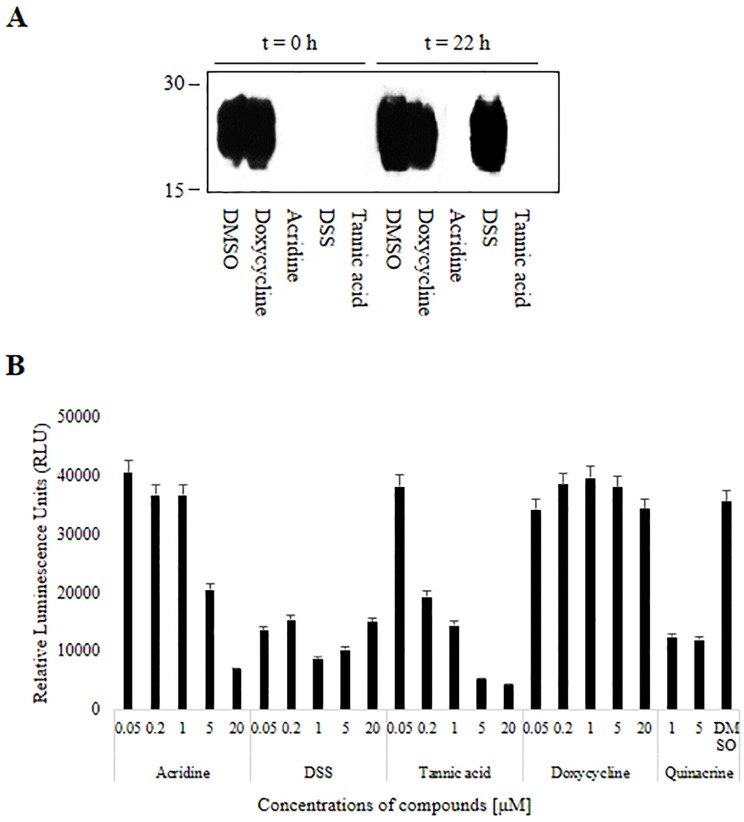
PrP^res^ in RT-QuIC products shown by western blot and MDS. PrP^res^ was detected in reaction products after application of compounds to RT-QuIC reactions at 0 h or 22 h (A). Luminescence as a marker of PrP^res^ formation is shown for each compound and concentration (B). Each compound was tested in duplicate in 3 independent experiments. Each value represents the mean and standard deviation; *P* < 0.05 and representative blots are shown.

To exclude the possibility that compounds were affecting the ThT fluorescence dye rather than substrate PrP^C^ or PrP aggregates, RT-QuIC products were collected from wells after the last reaction and PrP^res^ was detected using western blotting. PrP^res^ bands were not detected in RT-QuIC products from reactions that included acridine, DSS, or tannic acid at 0 h (Fig 3A, t = 0 h). PrP^res^ was not detected in products from reactions that included acridine, DSS, or tannic acid, whereas PrP^res^ was generated in wells with DMSO and doxycycline. Thus, we concluded that acridine, DSS, and tannic acid inhibited prion propagation in the RT-QuIC reaction and prevented the generation of PrP^res^. Additionally, PrP^res^ bands were not detected in RT-QuIC products from reactions that included acridine and tannic acid at 22 h, whereas these bands were detected in products from reactions that included DSS at 22 h (Fig 3A, t = 22 h). DMSO and doxycycline controls showed consistent amounts of PrP^res^. Western blotting results were similar to RT-QuIC results. We therefore confirmed that flat or reduced fluorescence in our RT-QuIC results was related to effects of the compounds on PrP^C^ or PrP^res^.

### Dye-independent conversion screening

To exclude the possibility that compounds were affecting the ThT fluorescence (i.e., ThT quenching) rather than substrate PrP^C^ or PrP^res^ aggregates, we performed a MDS enzyme-linked immunosorbent assay to quantify PrP^res^ formation in a dye-independent conversion assay. PrP^res^ was reduced by acridine and tannic acid in concentration-dependent manners from 0.05 to 20 μM. DSS also reduced PrP^res^ at all concentrations tested. The IC_50_ values for acridine, tannic acid, and DSS were 5 μM, 0.2 μM, and 0.05 μM, respectively. Quinacrine produced a positive result and doxycycline produced a negative result in this assay (Fig 3B). Accordingly, acridine, DSS, tannic acid, doxycycline, and DMSO produced consistent results in RT-QuIC (t = 0 h) and in the present assay. Therefore, direct ThT quenching by the test compounds was unlikely in this study.

### PrP^Sc^-infected cell screening

**Fig 4:**
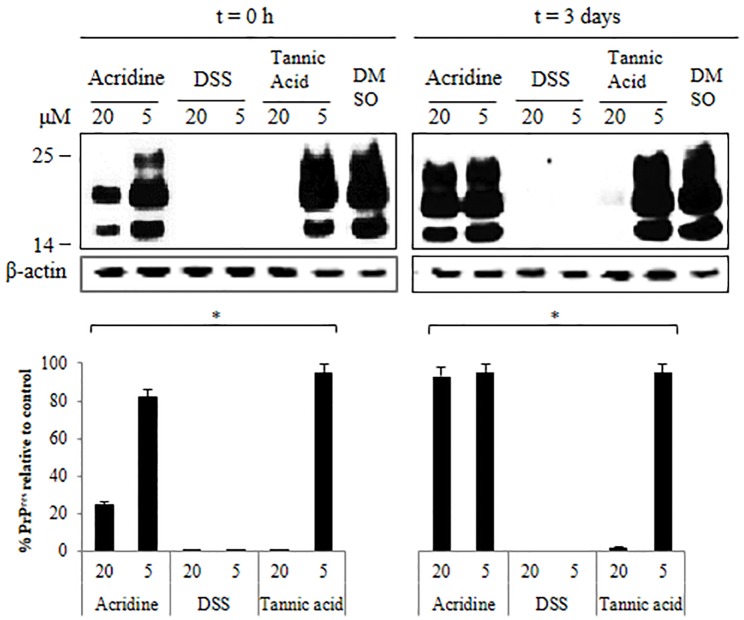
Western blots of PrP^res^ in cell screening. PrP^res^ after cell screening with test compounds and subsequent cell lysis for western blotting. Each compound was tested in duplicate in 3 independent experiments. The relative band densities are shown as the volume intensity/mm^2^ relative to the β-actin band density. Each value represents the mean and standard deviation; *P* < 0.05 and representative blots are shown.

Acridine, DSS, and tannic acid were applied to PrP^Sc^-infected cells at different concentrations (5 and 20 μM) at t = 0 h or t = 3 days. Relative amounts of PrP^res^ produced in cells treated with compounds were very similar between the 2 time intervals. Large PrP^res^ inhibitory effects were observed in response to 5 and 20 μM of DSS, while only 20 μM tannic acid produced a significant inhibitory effect in both experiments. Acridine only showed a partial inhibitory effect at 20 μM in the 0 h experiment (Fig 4, t = 0 h and t = 3 days). The inhibitory effects of acridine, dextran, and tannic acid in RT-QuIC and in cell-based screening are summarized in Table 2.

**Table 2:** Summary for anti-prion activities in RT-QuIC and cell-based screening.

Compounds	RT-QuIC		Cell-based assay	
	Treatment t = 0 h	Treatment t = 22 h	Treatment t = 0 h	Treatment t = 3 days
Acridine	+++	+++	+^a^	-
DSS	+++	-	+++	+++
Tannic acid	+++	+++	++^b^	++^b^

^a^Partial activity at high treatment only

^b^Full activity at high treatment only

## Discussion

A majority of anti-prion compounds have been confirmed *in vitro* and to some extent *in vivo* [6, 8]. However, compounds can affect a variety of factors and components in cells, including DNA, protein, and metal/non-metal ions. Therefore, some compounds may not directly inhibit the conversion of PrP^C^ or reduce the amplification of PrP^res^, but may have indirect effects on other cellular factors. Alternatively, the RT-QuIC assay has a relatively simple reaction environment. In the present study, possible environmental influences (especially ThT quenching by compounds) were in part excluded by performing follow-up western blotting of RT-QuIC reaction products and MDS. However, it is still unclear whether acridine inhibited PrP^res^ and quenched ThT at the same time. It has been reported that, in dye-dependent seeded aggregation assays, low molecular weight substances can interfere with the fluorescence of dye compounds bound to amyloid fibrils [27]. Indeed, some compounds have been posited to compete with ThT for the same binding site on fibrils rather than directly inhibit the fibrilization process. Other studies have similarly described the interference of small molecules with ThT fluorescence during fibril formation [28, 29]. To address this issue, we performed a dye-independent conversion assay in order to determine whether the test compounds indeed inhibited fibrilization. We concluded that the compounds in this study were unlikely to produce ThT quenching.

The present study thus demonstrates that RT-QuIC allows anti-prion compounds to be tested easily, specifically, and reliably: first, RT-QuIC can test the ability of compounds to inhibit the conversion of PrP^C^ to PrP^res^ (i.e., RT-QuIC screening after compound application at 0 h). Acridine, DSS, and tannic acid clearly inhibited the conversion of PrP^C^ to PrP^res^. Whether these compounds induce the inactivation of hot spot regions of PrP^C^ related to structural conversion or inactivate small amounts of PrP^Sc^ in brain seed should be confirmed in future studies. Second, RT-QuIC can determine the ability of compounds to eliminate or reduce PrP^res^ (i.e., RT-QuIC screening after compound application at 22 h/post-saturation). Acridine and tannic acid dramatically reduced PrP^res^ aggregates in this paradigm; however, whether these compounds target the structural unfolding of PrP^res^ or render pre-formed aggregates more PK-sensitive is an important topic for further study. Schmitz and colleagues suggested that their target compound bound seed particles in the RT-QuIC process and thus inhibited the transformation and aggregation steps [30]. Such a mechanism may be considered in the context of our findings.

It is important to note that, in our cell-based assays, the amount of PrP^res^ was not determined at baseline or other pre-treatment time points, such that we could not guarantee a zero-level of PrP^res^ in our t = 0 experiments. Treatment prior to the 24 h pre-incubation was not an option because immature cells are especially sensitive to non-toxic levels of test compounds and DMSO. Conversely, in cases of infection with PrP^Sc^ after compound treatment, a sufficient concentration of PrP^res^ could not be ensured with short passage periods. All of these issues can be addressed using RT-QuIC; the absence or presence of PrP^res^ can be confirmed by monitoring fluorescence during each reaction stage.

Given that amounts of PrP^res^ could not be monitored in cells, we tried to mimic different amounts of PrP^res^ in our RT-QuIC reactions by manipulating treatment times. We hypothesized that treatment at t = 0 h might permit compounds to affect the conversion of PrP^C^ to PrP^res^ easily in our reactions. Contrary to our expectation, relative amounts of PrP^res^ were similar between the 2 time conditions (t = 0 and 3 days) in DSS and tannic acid. However, the activity of acridine was higher when applied at t = 0 h than 3 days at 20 μM. It can be considered that acridine far reduced PrP^res^ by inhibiting the conversion of PrP^C^. However, there is no other existing evidence to support this activity given the past difficulty of monitoring PrP^res^ in cells. Alternatively, our observation may have been due to a longer incubation period in the 0 h treatment condition (total incubation period = 6 days) than in the 3 days treatment condition (total incubation period = 3 days). Notably, several laboratories have employed an incubation period of 8 days or more in cell screening assays [9, 10]. Therefore, some anti-prion compounds may show activity when applied for longer incubation periods than reported.

While the inhibitory effect of acridine was weak in the cell screening assay, it had the greatest effect in the RT-QuIC screening assay. If only cell screening was used for drug development, acridine would be excluded from subsequent studies, whereas its activity in the RT-QuIC assay would qualify it for future screening and indicate the potential for multiple mechanisms of action. Tannic acid showed a delayed effect on the degradation of PrP^res^ relative to acridine in RT-QuIC screening. This result suggests that the ability of acridine to degrade PrP^res^ was much greater than that of tannic acid. DSS showed powerful anti-prion activity and minimal cytotoxicity in cell screening; however, it only showed inhibitory activity in RT-QuIC screening at t = 0 h. The observed differences for DSS may be evidence that DSS has a great inhibitory effect and preferentially block the conversion of PrP^C^. Therefore, the mechanism of action of DSS is likely related to its effects on the generation of PrP^res^.

We can also infer possible mechanisms of action for acridine, DSS, and tannic acid with reference to other studies. A previous study used Fourier transform infrared spectroscopy and hydrogen-deuterium exchange to demonstrate structural differences between PrP^Sc^ derived from different strains and PrP^res^ generated by *in vitro* shaking, although the PK-resistant bands on western blotting were similar [31, 32]. The ScN2a cell line used in this study was infected with the RML strain, and RT-QuIC was based on *in vitro* shaking. Hence, it can be speculated that acridine was more effective at inhibiting PrP^res^ structures derived from the RT-QuIC assay than PrP^Sc^ structures from the strain in ScN2a cells. Additionally, it has been reported that unknown cofactors used for the conversion of PrP^C^ to PrP^Sc^ may be reduced during the PMCA reaction [33]. Another study showed that phosphatidylethanolamine was required as a cofactor for the amplification of PrP^Sc^ by a protein misfolding cyclic amplification [34]. One possible interpretation of our study is therefore that the drug performance of acridine may be interrupted by the presence of cofactors in cell screening. Alternatively, the activity of tannic acid was independent of the origin of PrP^Sc^ structures and cofactors, and consistent across both screening methods. Hence, tannic acid may have multiple mechanisms of action and be a candidate for advancement in future drug development studies.

Ideally, results from both RT-QuIC and cell screening should be consistent; however, we do not believe that it is necessary for these results to agree. Each screening method considers different mechanisms of action that are helpful for the further validation and understanding of anti-prion compounds. Of note, results from cell screening assays have not always been consistent with *in vivo* results. Many anti-prion compounds have only been studied *in vivo* as therapeutic agents given that (1) it is difficult to distinguish prophylactic versus therapeutic activity *in vitro*, (2) changes in PrP^res^ concentration are not easily monitored *in vitro*, and (3) it is difficult to determine whether a given cell incubation stage is suitable for prophylactic or therapeutic study. We suggest that RT-QuIC can be a preferable tool for the discovery of anti-prion compounds because of its versatility (for determining prophylactic versus therapeutic potential), reliability, and consistency. Thus, this study not only provides insights into prion biology, but also offers information for future studies and for prion disease therapeutic development.
